# Seed Priming Improved Antioxidant Defense System and Alleviated Ni-Induced Adversities in Rice Seedlings Under N, P, or K Deprivation

**DOI:** 10.3389/fpls.2020.565647

**Published:** 2020-09-03

**Authors:** Fahad Khan, Saddam Hussain, Sehrish Khan, Mingjian Geng

**Affiliations:** ^1^ Microelement Research Center, College of Resources and Environment, Huazhong Agricultural University, Wuhan, China; ^2^ Department of Agronomy, University of Agriculture, Faisalabad, Pakistan; ^3^ Department of Environmental Sciences, University of Peshawar, Peshawar, Pakistan

**Keywords:** seed priming, nutrient deprivation, nickel toxicity, oxidative damage, antioxidants

## Abstract

Excess nickel (Ni) concentration in the growing medium severely hampers the plant growth by disturbing oxidative metabolism and nutrient status. The present study was carried out to investigate the individual and interactive effects of Ni toxicity (0.25 mM NiSO4.6H2O) and nutrient deprivation (no-N, no-P, or no-K) on growth, oxidative metabolism, and nutrient uptake in primed and non-primed rice seedlings. Rice seed was primed with distilled water (hydropriming), selenium (5 mg L^-1^), or salicylic acid (100 mg L^-1^). The Ni toxicity and deprivation of N, P, or K posed negative effects on the establishment of rice seedlings. The shoot length and fresh biomass were severely reduced by Ni toxicity and nutrient stresses; the minimum shoot growth was recorded for rice seedlings grown under Ni toxicity and no-N stress. The Ni toxicity reduced the root fresh biomass but did not significantly affect the root length of N-deprived seedlings. The rice seedlings with no-P or no-K recorded similar root fresh biomass compared with those grown with sufficient nutrient supply. The Ni toxicity alone or in combination with nutrient stresses triggered the production of reactive oxygen species (ROS) and caused lipid peroxidation in rice seedlings. Among antioxidants, only glutathione reductase and vitamin E were significantly increased by Ni toxicity under different nutrient stress treatments. The Ni toxicity also reduced the concentrations of N particularly in shoot of rice seedlings. The N-deprived (no-N) seedlings recorded maximum Ni concentration in shoot, while K-deprived (no-K) seedlings showed higher Ni concentrations in root. Seed priming with selenium or salicylic acid was effective to alleviate the detrimental effects of Ni toxicity and/or nutrient stresses on rice seedlings. The better growth and greater stress tolerance of primed seedlings was coordinately attributed to lower ROS production, higher membrane stability, strong antioxidative defense system, and maintenance of mineral nutrient status.

## Introduction

Nickel (Ni) toxicity in plants is emerging as a worldwide problem threatening the agricultural sustainability. Generally, Ni is added into the environment by human activities like fossil fuel burning, metal mining, smelting, vehicle emissions, wastes disposal, and crop fertilization (Salt et al., 2000; Hussain et al., 2020a). Extremely high Ni concentrations in soil make the cultivatable land unfit for the cultivation of crops (Duarte et al., 2008). Higher range of Ni in the growing medium causes various changes in the different physiological and metabolic processes of plants, and leads to assorted toxicity indications (Kumar et al., 2007; Gajewska et al., 2009; Gajewska et al., 2013). Gajewska et al. (2013) found severe reductions in shoot and root growth of wheat under Ni stress, which were attributed restriction of cell division and elongation (Gajewska et al., 2009). Nickel stress may also reduce dry matter accumulation in different plant parts thus reduces the total plant biomass (Rao and Sresty, 2000; Pandey and Sharma, 2002; Rizwan et al., 2019).

Increasing evidences have suggested that the Ni toxicity in plants is also associated with the oxidative stress through increase in the production of reactive oxygen species (ROS) like hydrogen peroxide (H_2_O_2_), hydroxyl ion (OH^-^), and super and nitric oxides anions (Galan et al., 2001; Gajewska et al., 2006; Hao et al., 2006; Gajewska and Sklodowska, 2007). These ROS may damage plant cell membrane, proteins, DNA and lipids, and cause lipid per oxidation (Bal and Kasprzak, 2002; Khaliq et al., 2015; Chen et al., 2016; Hussain et al., 2020b). Rao and Sresty (2000) observed increased production of a lipid peroxidation [malondialdehyde content (MDA)] in Ni exposed pigeon pea plants. Maize exposure to Ni stress significantly increased H_2_O_2_ production and antioxidants activity in the leaves (Kumar et al., 2007). Nickel cannot directly induce the production of ROS because it is not a redox-active metal, but indirectly it may play vital role in activation of antioxidant enzymes (Pandey and Sharma, 2002; Gajewska and Sklodowska, 2005) like superoxide dismutase (SOD), peroxidase (POD), catalase (CAT), glutathione peroxidase (GPX), and glutathione reductase (GR) (Gajewska and Sklodowska, 2005). Conversely, Zhao et al. (2008) and Gajewska and Sklodowska (2007) observed that Ni stress decreased the activities of several antioxidant enzymes and triggered the ROS accumulation and oxidative stress.

Imbalanced nutrient uptake is also a major response of plants in Ni toxic conditions (Pandey and Sharma, 2002; Ouzounidou et al., 2006; Hussain et al., 2020a). Gajewska et al. (2013) found the decrease in the concentration of nutrients in Ni exposed wheat, as Ni toxicity restricts nutrient uptake and causes nutrient deficiency (Chen et al., 2009) ultimately leading to disturbed physiological and biochemical processes in plant (Gajewska et al., 2006; Rizwan et al., 2019).

Seed priming is a method that manages the level of hydration within seeds and regulates the metabolic events in seed required for germination. Earlier researches have demonstrated that germination, seedling vigor, and survival of rice seedlings were enhanced by seed priming under normal and adverse soil and climatic conditions (Jisha et al., 2013; Hussain et al., 2016a; Hussain et al., 2016b; Hussain et al., 2016c; Zheng et al., 2016). Selenium (Se) is an important element, reported for the detoxification of toxic heavy metals in plants (Hasanuzzaman et al., 2010; Zembala et al., 2010), while salicylic acid (SA) is a phenolic compound and considered as an important growth regulator in the plants (Hayat et al., 2010). The positive effects of both Se and SA in plants under different abiotic stresses have been well reported (Sun et al., 2010; Hayat et al., 2010; Hasanuzzaman et al., 2010; Cui et al., 2012; Kumar et al., 2012; Gajewska et al., 2013). In our recent investigations, we found that Se and SA priming enhanced the rice tolerance against different stress factors including chilling, submergence, nutrient deprivation, and lead toxicity (Hussain et al., 2016a; Hussain et al., 2016b; Hussain et al., 2016c; Khan et al., 2018). However, little work has been done on the role of seed priming in enhancing the plant tolerance against combined Ni toxicity and nutrient deprivation. It was hypothesized that the toxic effects of Ni will be different with the supply of N, P, or K, and that the behavior of primed and non-primed rice seedlings regarding growth and oxidative metabolism will be variable under these stress factors. The present study was carried out to investigate the individual and interactive effects of Ni toxicity and N, P, or K-deprivation on growth, ROS production, antioxidant defense system, and nutrient homeostasis in primed and non-primed rice seedlings grown in hydroponic culture experiment.

## Material and Methods

### Growth Conditions, Treatments, and Experimental Set Up

Inbred rice cultivar “Huanghuazhan” seedlings were cultivated in plastic pots containing nutrient solution (pH: 6.6 ± 0.2) in a controlled growth chamber. The conditions of the growth chamber during the course of study were set as; temperature (day/night): 30/25°C, light intensity: 25,000 Lx, light period: 12 h; and humidity: 60%. Seed was treated with distilled water (hydropriming; HP), selenium (Se: 5 mg L^-1^), and salicylic acid (SA: 100 mg L^-1^) following standard procedure (Hussain et al., 2015; Hussain et al., 2016a; Hussain et al., 2016b). The Ni stress was applied through 0.25 mM NiSO4.6H2O from the start of experiment. The nutrient treatments were divided into four different groups as, sufficient nutrient supply (All nutrient), no-N (N deprivation), no-P (P deprivation), and no-K (K deprivation). The Hoagland’s nutrient solution was used according to the recommendation of International Rice Research Institute (Yoshida et al., 1976), with some modifications as per treatment, as mentioned in our previous investigations (Hussain et al., 2016b; Khan et al., 2018). All the nutrients were refreshed after every alternate day. Plastic pots with 4 L of solution and a floating board with four separated sections (for different priming treatments) were used, and twenty seeds of each treatment were separately sown on the net attached with the floating board. All the treatments were arranged in completely randomized design (CRD) with three replications.

### Observations

Eighteen days old rice seedlings were harvested and morphological growth attributes viz., shoot length, root length and their fresh weights were recorded using at least five seedlings randomly selected from each replicate. The maximum shoot and root lengths of the rice seedlings were recording using measuring tape, while digital electric balance was used for recording fresh weight. Fresh leaf samples were stored in -80°C refrigerator for analysis of different biochemical attributes.

The H_2_O_2_ and MDA contents (µM g^-1^ fresh weight) in the leaves were determined by the procedure of Patterson et al. (1984) and Bailly et al. (1996), respectively. Whereas, commercial O2•− assay kit-A052, OH− assay kit-A018, XOD assay kit-A034, and MAO assay kit-A002 were used for determination of superoxide anion radical (O2•− as U g^-1^ protein) content, hydroxyl ion (OH− as U mg^-1^ protein) content, xanthine oxidase (XOD as U g^-1^ protein) activity, and monoamine oxidase (MAO as U mg^-1^ protein) activity in the rice leaves, respectively.

The antioxidant activities/levels of SOD (U mg^-1^ protein), CAT (U mg^-1^ protein), POD (U mg^-1^ protein), GR (U g^-1^ protein), GPX (U mg^-1^ protein), GSH (reduced glutathione; μM g^-1^ protein), Vc (vitamin C; μg mg^-1^ protein), and Ve (vitamin E; μg g^-1^ tissue fresh weight in rice were recorded using commercial kits A001, A007-2, A084-3, A062, A005, A006, A009, and A008, respectively (Hussain et al., 2016a; Hussain et al., 2016b; Khan et al., 2018; Hussain et al., 2020b). All the kits used in the present study were purchased from Nanjing Jiancheng Bioengineering Institute, China (www.njjcbio.com), and were strictly used as per manufacturer’s instructions. For determination of N, P, and K in shoots and roots, rice dry seedlings were digested with sulfuric acid. The N and P concentrations in the plant tissues were recorded by a continuous-flow injection analyzer, while K concentrations were analyzed using a flame photometer. For Ni determination, roots and shoots were digested in HNO3:HClO4 at 5:1 (v/v), and samples were analyzed using ICP-MS (Inductively coupled plasma mass spectrometry) technique.

### Statistical Analysis

The replicated data were analyzed using analysis of variance (ANOVA) through Statistix 8.1 software. The treatment means were compared according to Tukey’s HSD (P ≤ 0.05) test.

## Results

### Seedling Growth

**Figure 1:**
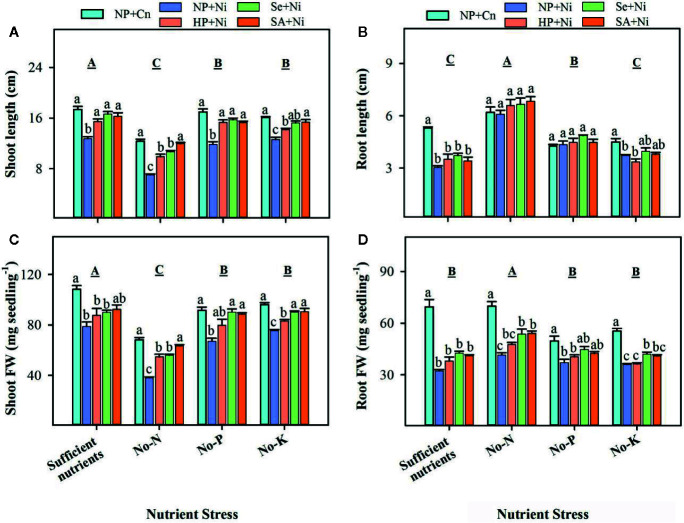
Shoot length **(A)**, root length **(B)**, shoot fresh weight **(C)**, and root fresh weight **(D)** of primed and non-primed rice seedlings as influenced by different nutrient stress treatments and Ni toxicity. Vertical bars above mean indicate standard error of three replicates. Small alphabetical letters (a, b, c…) above mean bars show the differences among treatments within a specific nutrient stress treatment, while the capital alphabetical letter (A, B, C…) show the difference among nutrient stress treatments. NP+Cn, no priming and no Ni toxicity; NP+Ni, no priming with Ni toxicity; HP+Ni, hydropriming and Ni toxicity; Se+Ni, selenium priming and Ni toxicity; SA+Ni, salicylic acid priming and Ni toxicity; No-N, no nitrogen; No-P, no phosphorus; No-K, no potassium.

Data regarding rice seedling growth as affected by Ni toxicity, seed priming and different nutrient stress treatments are shown in **Figure 1**. Compared with sufficient nutrient supply, shoot length, and shoot fresh weight were significantly (p < 0.05) reduced in no-N, no-P, and no-K treatments; the minimum shoot growth was noted in no-N treatment. Root length was considerably increased in no-P or no-N treatments, but no-K did not significantly (p > 0.05) affect the root length. Rice seedlings with no-N recorded significantly (p < 0.05) higher root fresh weight compared with all other nutrient stress treatments. Exposure of Ni toxicity in non-primed seedlings (NP + Ni) recorded significantly (p < 0.05) lower shoot length and shoot fresh weight, compared with NP+Cn under all the nutrient stress treatments. The Ni induced reductions in shoot growth were more apparent in no-N treatment. The Ni toxicity did not significantly (p > 0.05) affect the root length of rice in no-N or no-P treatments, but root length was significantly (p < 0.05) reduced by Ni toxicity in rice seedlings with sufficient nutrient supply. Root fresh weight was significantly (p < 0.05) reduced by Ni toxicity in all nutrient stress treatments. Seed priming was found to alleviate the detrimental effects of Ni toxicity particularly on shoot growth, therefore, Se+Ni, and SA+Ni treatments recorded significantly (p < 0.05) higher shoot length and shoot fresh weight under no-N, no-P, and no-K treatments, compared with NP+Ni. Seed priming didn’t significantly (p > 0.05) affect the root length; however, root fresh weight of rice in Se+Ni and SA+Ni under no-N, and Se+Ni under no-K was significantly (p < 0.05) higher with respect to NP+Ni (**Figure 1**).

### Accumulation of ROS and Lipid Peroxidation Rate

**Figure 2:**
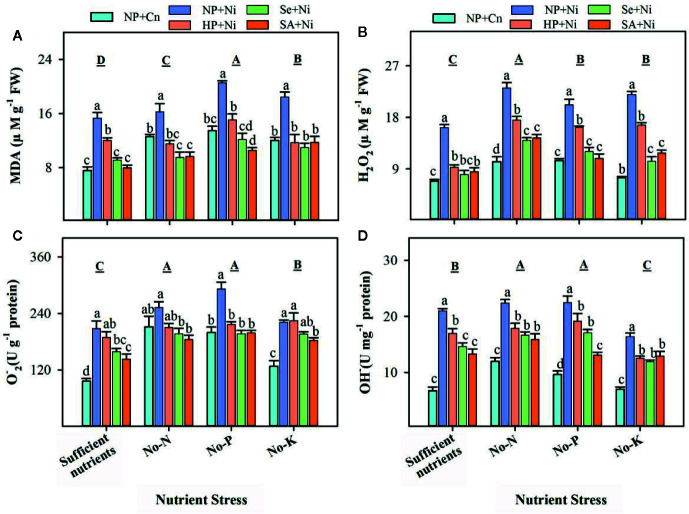
The accumulation of reactive oxygen species and lipid peroxidation rate in the leaves of primed and non-primed rice seedlings as influenced by different nutrient stress treatments and Ni toxicity. **(A)** Malondialdehyde (MDA) content, **(B)** hydrogen peroxide (H_2_O_2_) content, **(C)** superoxide anion radical (O_2_^•^−) content, **(D)** hydroxyl ion (OH^−^) content. Details on statistical analysis and treatments are given in **Figure 1**. “The 1 U of OH^-^ was the amount required to reduce 1 M of H_2_O_2_ in the reaction mixture per minute at 37°C”, while “1 U of $O_{2}^{-}$ was equivalent of the value required to inhibit superoxide anion by 1 mg of Vc for 40 min at 37°C”.

Pronounced variations in the accumulation of ROS and lipid peroxidation rate in non-primed and primed rice seedlings were recorded under the influence of Ni toxicity and different nutrient stress treatments (**Figure 2**). The rate of lipid peroxidation (MDA contents) and the accumulation of O2•−, OH–, and H_2_O_2_ were significantly (p < 0.05) increased under no-N, no-P, and no-K treatments compared with sufficient nutrient supply (**Figure 2**). Nickel toxicity also significantly (p < 0.05) enhanced the accumulation of ROS and MDA contents in rice leaves regardless of the nutrient stress treatment. Seed priming was effective in decreasing ROS accumulation as well as lipid peroxidation in rice leaves under Ni toxicity and different nutrient stress treatments (**Figure 2**). Therefore, the accumulations of ROS and MDA were significantly (p < 0.05) lower in HP+Ni, Se+Ni, SA+Ni compared with NP+Ni under all nutrient stress treatments (**Figure 2**).

### Activities of Xanthine Oxidase and Monoamine Oxidase

**Figure 3:**
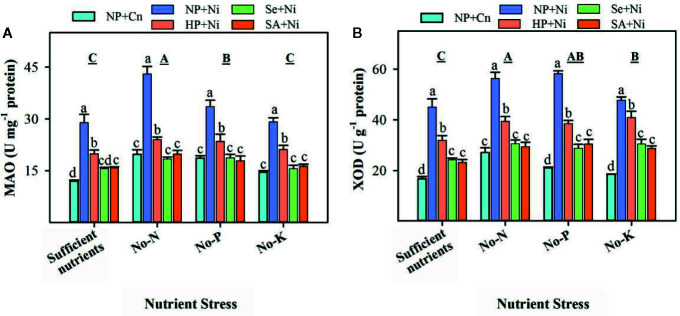
Activities of monoamine oxidase (MAO) **(A)** and xanthine oxidase (XOD) **(B)** in the leaves of primed and non-primed rice seedlings as influenced by different nutrient stress treatments and Ni toxicity. Details on statistical analysis and treatments are given in **Figure 1**. “For MAO, 1 U was defined as the amount of enzyme that increased the absorbance by 0.01 at 37°C in 1 hour; while “for XOD, it was defined as 1 g of protein required to transform 1 μM of hypoxanthine to xanthine in 1 min at 37°C”.

The activities of XOD and MAO in leaves of non-primed and primed rice seedlings significantly (p < 0.05) varied in response to Ni toxicity and nutrient stresses (**Figure 3**). Compared with sufficient nutrient supply, MAO activities were significantly (p < 0.05) enhanced in no-N and no-P treatments, while XOD activities were increased significantly (p < 0.05) under the deprivation of N, P, or K (**Figure 3**). Among different nutrient stress treatments, the highest MAO and XOD activities were observed in rice seedlings grown in no-N. Effects of Ni toxicity were apparent in enhancing the MAO and XOD activities; NP+Ni recorded significantly higher activities of both these enzymes compared with NP+Cn under different nutrient stress treatments (**Figure 3**). The Ni-induced increases in MAO and XOD activities were more in no-P or no-N treatments. The activities of XOD and MAO in rice seedling were significantly (p < 0.05) decreased by seed priming. Compared to NP+Ni, all seed priming treatments significantly decreased the MAO and XOD activities in leaves under different nutrient stress treatments; however, Se+Ni and SA+Ni treatments were statistically (p > 0.05) similar, and were more effective than HP+Ni (**Figure 3**).

### Enzymatic Antioxidants

**Figure 4:**
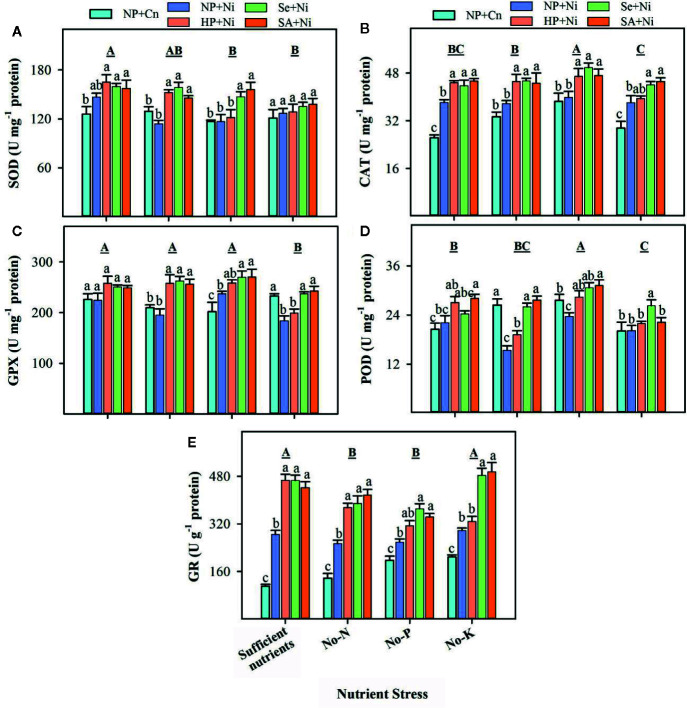
Activities of various enzymatic antioxidants in the leaves of primed and non-primed rice seedlings as influenced by different nutrient stress treatments and Ni toxicity. **(A)** Superoxide dismutase (SOD), **(B)** catalase (CAT), **(C)** glutathione peroxidase (GPX), **(D)** peroxidase (POD), and **(E)** glutathione reductase (GR). Details on statistical analysis and treatments are given in **Figure 1**. One unit for different antioxidant enzymes was defined as follows; “I U of SOD activity was the amount of enzyme required to decrease the reference rate to 50% of maximum inhibition; 1 U of POD activity was defined as the amount of enzyme necessary for the decomposition of 1 μg substrate in 1 min at 37°C; 1 U of CAT activity was defined as the amount of enzyme required to decompose the 1 μM H_2_O_2_ in 1 second at 37°C; I U of GPX activity was the amount of enzyme required to oxidize 1 μM GSH in 1 minute at 37°C; 1 U of GR activity was defined as the amount of enzyme depleting 1 mM NADPH in 1 min”.

Data on the activities of enzymatic antioxidants in non-primed and primed rice seedlings in response to Ni toxicity and nutrient stresses are presented in **Figure 4**. Compared with sufficient nutrient supply, SOD activities remained unaffected in no-N, but significantly (p < 0.05) decreased in no-P or no-K treatments. The activities of CAT were significantly (p < 0.05) higher in no-P, but remained unchanged in no-N or no-K, compared with sufficient nutrient supply. The GPX and POD activities were significantly (p < 0.05) decreased in no-K, but remained unchanged in no-N with respect to sufficient nutrient supply. The activities of GR were significantly (p < 0.05) decreased in no-N or no-P treatments. The Ni toxicity considerably affected the activities of enzymatic antioxidants, however, such effect varied with enzyme and nutrient stress. The SOD activity was unaffected, while GR activity was significantly (p < 0.05) increased in NP+Ni than NP+Cn, under all nutrient stress treatments. The CAT activity was significantly (p < 0.05) higher in NP+Ni under no-K or sufficient nutrient supply, while remained statistically similar (p > 0.05) to NP+Cn under no-N or no-P. Conversely, the NP+Ni significantly (p < 0.05) decreased the activity of POD under no-N or no-P, but it did not affect POD under no-K or sufficient nutrient supply compared to NP+Cn. The GPX activity was increased in NP+Ni under no-P, decreased under no-K, while did not change under no-N or sufficient nutrient supply, compared to NP+Cn (**Figure 4**). Seed priming (Se+Ni and SA+Ni) significantly (p < 0.05) increased the CAT and GR activities under different nutrient stress, compared with NP+Ni. The SOD, POD, and GPX activities were also significantly (p < 0.05) higher in both Se+Ni and SA+Ni treatments under no-N or no-P, compared with NP+Ni. Under no-K, Se+Ni recorded higher activities of GPX and POD compared with NP+Ni (**Figure 4**).

### Non-Enzymatic Antioxidants

**Figure 5:**
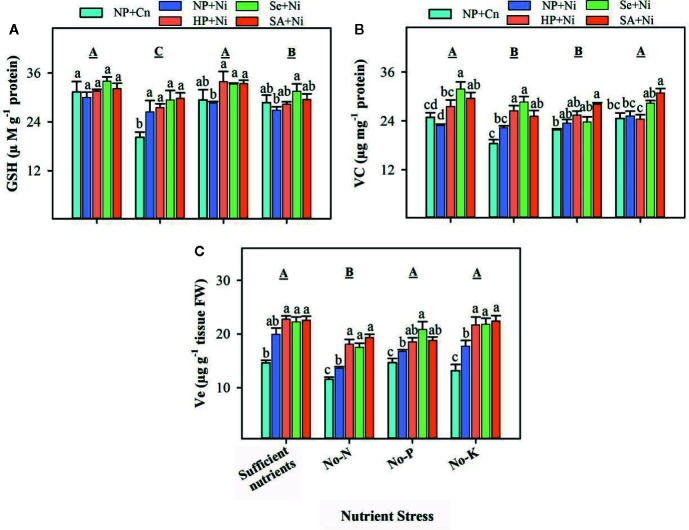
The levels of various non-enzymatic antioxidants in the leaves of primed and non- primed rice seedlings as influenced by different nutrient stress treatments and Ni toxicity. **(A)** Reduced glutathione (GSH), **(B)** vitamin C (Vc), **(C)** vitamin E (Ve). Details on statistical analysis and treatments are given in **Figure 1**.

Compared with sufficient nutrient supply, the GSH, Vc, and Ve contents in rice seedlings were significantly (p < 0.05) reduced in no-N (**Figure 5**). The no-P didn’t significantly (p > 0.05) affect GSH and Ve, but significantly (p < 0.05) declined the Vc concentration in rice leaves. The no-K significantly (p < 0.05) decreased the GSH content, but did not affect (p > 0.05) Vc and Ve content compared with sufficient nutrient supply (**Figure 5**). The Ni toxicity did not significantly (p > 0.05) alter the levels of GSH and Vc in all the nutrient stress treatments expect for GSH content in no-N. The Ve content was significantly (p < 0.05) increased by Ni stress (NP+Ni) under the deprivation of N, P, or K. Seed priming enhanced or at least maintained the levels of non-enzymatic antioxidants in the leaves of rice seedlings. All the seed priming treatments significantly (p < 0.05) enhanced the GSH content in no-P treatment, and Ve content in no-N and no-K treatments. Seed priming also significantly (p < 0.05) increased the Vc under sufficient nutrient supply, but did not change it under no-P, compared to NP+Ni. Significantly higher Vc contents in HP+Ni and Se+Ni under no-N, and in SA+Ni under no-K were also observed compared with NP+Ni (**Figure 5**).

### Macro-Nutrient Concentrations in Root and Shoot Tissues

**Figure 6:**
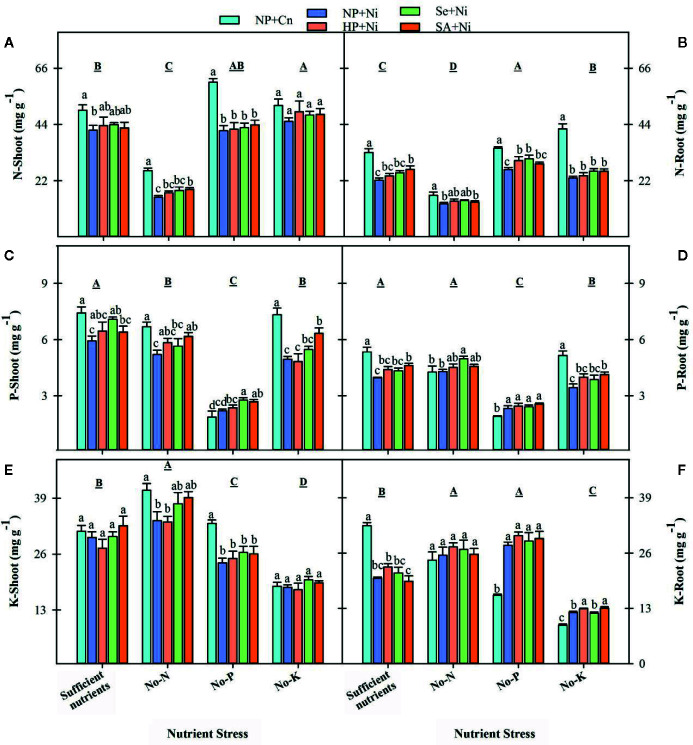
Shoot and root concentrations of nitrogen **(A, B)**, phosphorus **(C, D)**, and potassium **(E, F)** in primed and non-primed rice seedlings as influenced by different nutrient stress treatments and Ni toxicity. Details on statistical analysis and treatments are given in **Figure 1**.

The N concentrations in root and shoot of rice were significantly (p < 0.05) declined in no-N, but increased in no-K, compared with sufficient nutrient supply (**Figure 6**). The P-deprivation did not significantly (p > 0.05) affect the shoot N concentrations, but significantly (p < 0.05) increased the root N concentrations compared with sufficient nutrient supply. The P concentrations of both shoot and root in rice seedling were significantly (p < 0.05) decreased in no-P and no-K treatments, the shoot P concentrations were also significantly (p < 0.05) decreased in no-N with respect to sufficient nutrient supply. The shoot K concentration was significantly (p < 0.05) increased in no-N, but it was reduced in no-P or no-K treatments. The root K concentration was increased in no-N or no-P treatment, but significantly reduced in no-K compared with sufficient nutrient supply (**Figure 6**). The Ni toxicity significantly reduced both root and shoot N concentrations under all nutrient stress treatments, except for shoot N concentration in no-K treatment, compared with NP+Cn. The shoot and root P concentrations were significantly reduced by Ni toxicity in no-K and sufficient nutrients treatment. The Ni toxicity (NP+Ni) significantly decreased the shoot K concentrations in no-N or no-P treatments, while significantly increased root K concentrations in no-P or no-K treatments, compared with NP+Cn (**Figure 6**). Seed priming had positive effect on the uptake of primary macro-nutrients under different stress treatments. Compared with NP+Ni, seed priming treatments (NP+Ni, Se+Ni, SA+Ni) recorded statistically similar or higher concentration of N, P, and K in root and shoot tissues of rice, under different nutrient stress treatments (**Figure 6**).

### Nickel (Ni) Concentrations in Root and Shoot Tissues

**Figure 7:**
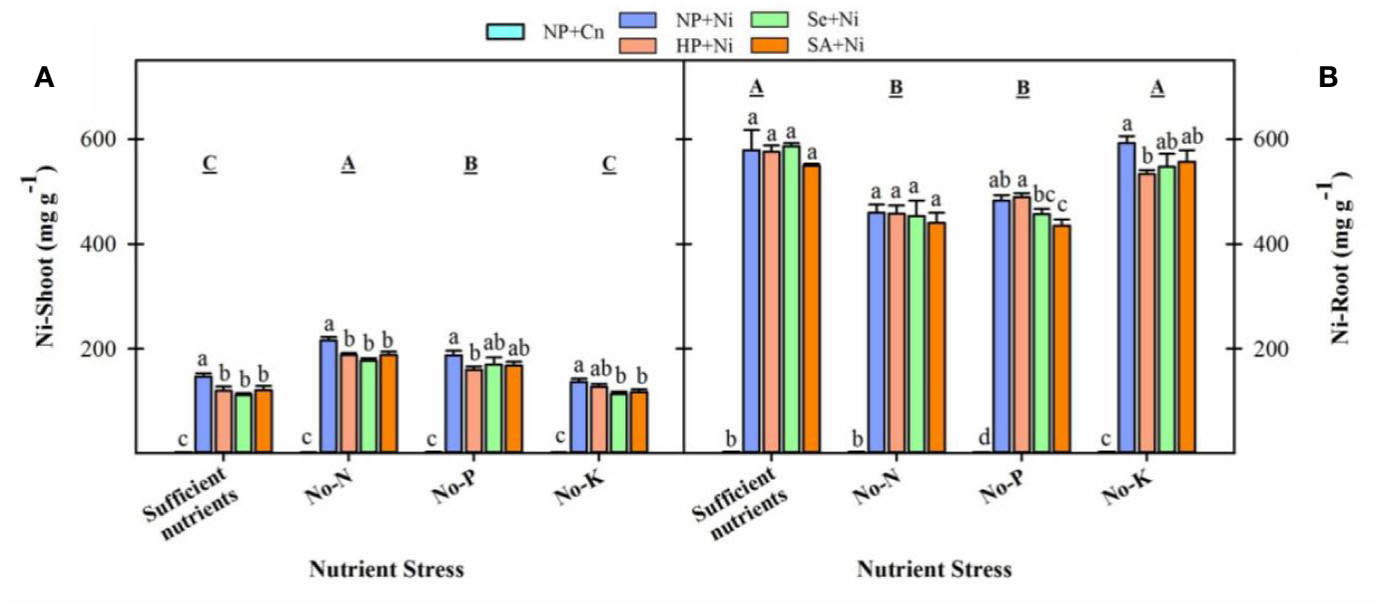
Shoot **(A)** and root **(B)** concentrations of nickel in primed and non-primed rice seedlings as influenced by different nutrient stress treatments and Ni toxicity. Details on statistical analysis and treatments are given in **Figure 1**.

Among different nutrient stress treatments, the Ni shoot concentrations were maximum in no-N treatment followed by no-P, while Ni root concentrations were maximum in rice seedlings with sufficient nutrient supply (**Figure 7**). The Ni concentration of shoot and root were significantly increased with application of Ni under all nutrient stress treatments. Seed priming (Se+Ni and SA+Ni) were effective to significantly decrease shoot Ni concentrations compared to NP+Ni under all nutrient stress treatments except no-P. The root Ni concentrations remained statistically similar in both primed and unprimed seedlings; only SA+Ni and HP+Ni recorded significantly lower root Ni concentrations in no-P and no-K treatments, respectively compared to NP+Ni (**Figure 7**).

## Discussion

Under natural conditions, multiple abiotic stresses often occur at a same time, and alter the growth of plants (Walter et al., 2012; Hussain et al., 2016b), however, the responses of plants to individual and combined stresses might be variable at physiological, biochemical, and molecular levels (Hussain et al., 2016b; Hussain et al., 2020b). The present study investigated the effects of different seed priming treatments on the growth, oxidative metabolism, and nutrient uptake in rice seedlings under individual and combined exposure of Ni toxicity and N, P, or K deprivation.

### Ni Toxicity as Well N, P, or K Deprivation Triggered the Oxidative Damage in Rice Seedlings

The Ni toxicity as well as different nutrient stresses disrupted the oxidative metabolism in rice seedlings, and thus enhanced the production of ROS in rice leaves (**Figure 3**). The higher production of ROS also triggered the accumulation of MDA content and oxidative damage in rice leaves (**Figure 3A**) and ultimately reduced the growth. Although, the interactive influence of Ni and nutrient deprivation on oxidative metabolism in plants is rarely known previously, nevertheless, several studies have documented that individual application of Ni toxicity or N, P, K deficiency caused severe oxidative damage in various plants. For instance, Shin et al. (2005) documented that N, P, or K deprivation increased the accumulation of ROS in arabidopsis roots. Gajewska et al. (2006) reported that Ni toxicity in plants caused oxidative stress in wheat. In pigeon pea, Ni toxicity from 0.5 to 1.5 mM promoted the accumulation of O2•−, OH–, H_2_O_2_ and MDA content in both shoot and root (Rao and Sresty, 2000). Many enzymatic sources like XOD, MAO, and NADPH oxidase have also been reported to control the production of ROS under stress (Hussain et al., 2016b), in addition to non-enzymatic sources of ROS production (limitation of CO_2_ fixation, photorespiration etc.). Hao et al. (2006) described that the Ni stress regulated the NADPH oxidase activity, which induced ROS production in the roots of 5-day-old wheat seedlings. In the present study, the activities of XOD and MAO in rice leaves were enhanced under Ni toxicity and different nutrient stresses, and activities of both these enzymes were concomitant with ROS accumulation (**Figures 2** and **3**), which indicated that XOD and MAO also contributed in the production of ROS under stress conditions. Plants overcome the excessive production of ROS through coordinated action of different enzymatic (such as SOD, CAT, POD, GPX, GR, and GST) and non-enzymatic antioxidant (such as GSH, Vc, and Ve) (Gill and Tuteja, 2010; Hasanuzzaman and Fujita, 2011; Chen et al., 2016; Hussain et al., 2016a; Hussain et al., 2016b; Hussain et al., 2020b). In the present study, the responses of different antioxidants varied with the enzyme and stress conditions. For instance, compared with NP+Cn, the SOD activity in NP+Ni was generally unaffected, the CAT activity was triggered under no-K, while the POD activity in NP+Ni was decreased under no-N or no-P. The lower activities of CAT and POD enzymes in NP+Ni under P or N-deprivation were well linked with higher oxidative damage in these treatments (**Figures 2** and **4**). Previously, differential responses of these antioxidant enzymes were observed under Ni toxicity by different researchers. For example, Gajewska and Sklodowska (2007) found that SOD and CAT activities were markedly reduced in wheat in response to 100 µM Ni treatment. Likewise, Pandey and Sharma (2002) reported that exposure of cabbage to 0.5 mM Ni for 8 days reduced the CAT and POD activities. Papadopoulos et al. (2007) found that exposure of *Hydrocharis dubia* to 0.5, 1, 2, 3, 4 mM Ni for 3 days caused reductions in the activities of SOD, POD, and CAT in leaves. In disparity, Rao and Sresty (2000) stated that activities of SOD and POD were increased at 0.5 mM Ni concentration while CAT activity was declined in 6-day-old pigeon pea seedlings. In the present study, GR activity was considerably enhanced by Ni toxicity under different nutrient stress treatments, suggesting that this enzyme was more responsive to Ni stress. Similar results regarding GR activity were also found by Rao and Sresty (2000) in pigeon pea.

Along with enzymatic antioxidants, the significant role of GSH, Vc, and Ve in the tolerance of plants to abiotic stresses has been well proven (Foyer and Noctor, 2005; Gill and Tuteja, 2010; Hussain et al., 2016a; Hussain et al., 2016b; Hussain et al., 2020b). In this study, variable response of Ve, GSH and Vc in rice leaves were detected under Ni toxicity and nutrient deprivation (**Figure 5**). Among different nutrient stresses, the accumulations of all these non-enzymatic antioxidants were significantly decreased in no-N treatments, indicating that N availability is critical for synthesis and accumulation of these molecules. Exposure of Ni stress generally triggered the Ve content, but did not alter the Vc and GSH content under all the nutrient treatments except GSH under N-deprivation (**Figure 5**). Although GR was considerably increased by Ni toxicity, minimal effect of Ni on GSH content suggests that biosynthesis of GSH might have been limited by Ni stress. Several researchers have observed that the Ni toxicity decreased the Vc and GSH concentrations, which may cause higher oxidative stress in plants (Kukkola et al., 2000; Rao and Sresty, 2000).

### Seed Priming Triggered the Antioxidant Defense System and Alleviated the Stress-Evoked Adversities in Rice Seedlings

Seed priming significantly decreased the accumulation of ROS and rate of lipid peroxidation (**Figure 2**), which indicated that oxidative stress and seedling damage, induced by Ni toxicity and/or nutrient deprivation, were effectively assuaged by seed priming. Various researches on different abiotic stresses have reported the similar priming-induced effects on ROS accumulation and MDA content (Jisha et al., 2013; Hussain et al., 2016a; Hussain et al., 2016b; Zheng et al., 2016; Hussain et al., 2018; Hussain et al., 2020b). The activities of ROS producing enzymes such as, XOD and MAO were also restricted by seed priming (**Figure 3**). The SA and Se priming recorded higher activities/levels of SOD, CAT, POD, GR, GSH, Vc, and Ve under Ni toxicity and nutrient deprived conditions over no priming (**Figures 4** and **5**). The significantly higher activities/contents of these antioxidants in primed rice seedlings were associated with the lower buildup of ROS in rice leaves. All the antioxidants triggered by seed priming were effective in controlling the ROS, which are very important for inducing tolerance to abiotic stresses (Gill and Tuteja, 2010; Miller et al., 2010). Previously, many studies revealed that seed priming enhanced antioxidant levels in seed which help to cope with stress induced adversities after germination (Bailly et al., 2008; Khaliq et al., 2015; Hussain et al., 2016a; Hussain et al., 2016b).

### Ni Toxicity Disrupted the Mineral Nutrient Status of Non-Primed Rice Seedlings Under Different Nutrient Stress Treatments

The Ni toxicity is well known to inhibit the nutrient (such as N, P, and K) uptake in plants (Pandey and Sharma, 2002; Ouzounidou et al., 2006). In the present study, Ni toxicity caused macro-nutrients deficiency in rice seedlings; however, the effects varied with plant parts and nutrient stress treatments (**Figure 6**). The Ni induced reductions in N, P, and K concentrations were more for shoot compared with root particularly under no-N, indicating that Ni stress mainly limited the translocation of these elements. These findings are in agreement to Brune and Deitz (1995) who also observed that Ni toxicity caused P and K deficiency in leaves and roots. Athar and Ahmad (2002) reported that Ni toxicity reduced root and shoots N content in mungbean and chickpea plants, and P content in *Helianthus annus* and *Hyptis suaveolens*, and these effects were attributed to increased activity of acid phosphatase and ATPase under Ni toxicity (Pillay et al., 1996). Heavy metals including Ni alter the functions and structure of membrane (Gajewska et al., 2006), which decrease the nutrient uptake and translocation to root and shoot. Seed priming was found to improve the uptake of N, P, and K under Ni toxicity. The growth of primed rice seedlings was significantly higher than non-primed rice seedlings (**Figure 1**), while the nutrient concentrations were generally similar or higher in primed rice seedlings (**Figure 6**). When considering the overall nutrient content per seedlings, primed rice seedlings were able to accumulate higher quantity of nutrients in its plant parts. Previously, several researchers (Gunes et al., 2007; Shah et al., 2012; Shah et al., 2013; Ahmad et al., 2015; Khan et al., 2018) have concluded that seed priming improved uptake and translocation of mineral nutrients in plants. Ahmad et al. (2015) noted improved root and shoot concentrations of N, P, and K by seed priming with ascorbic acid and hydrogen peroxide, and attributed it to vigorous and well developed root system.

### Ni Accumulation in Rice Seedlings Varied With Nutrient Stress Treatment and Seed Priming

Exposure of Ni toxicity triggered its concentrations in both plant parts of rice seedlings, nevertheless, the Ni concentrations varied greatly with N, P, or K deprivation and seed priming (**Figure 7**). Differences were also apparent between plant parts, as more Ni was accumulated in the root, which is the common tolerance mechanism of different plant species including rice to heavy metals. Interestingly, the shoot Ni concentrations were higher in no-N or no-P treatments, while root Ni concentrations were higher in treatments with no-K or sufficient nutrient supply. It might be suspected that the better seedling performance and higher tolerance in treatments with no-K or sufficient nutrient supply might also be due to the lower Ni translocation. In the present study, the total shoot and root Ni concentrations were recorded, therefore, it is not known whether N, P, or K- deprivation altered the Ni distribution between vacuolar compartments and the other parts of the cell. In the future studies, determining the effect of nutrient deprivation/deficiency on Ni concentration in different cellular compartments will provide a better understanding. The seed priming treatments generally recorded similar root concentrations, but lower shoot concentrations than non-primed treatment. Overall, the lower Ni accumulations in primed rice seedlings were reflected in the maintenance of growth characteristics and nutrient’s status on the level close to the control (**Figures 1**, **6**, and **7**). The seed priming with Se and SA was found to be more effective against Ni toxicity than hydropriming. By now, a number of evidences have revealed the effectiveness of Se (Hasanuzzaman et al., 2010; Sun et al., 2010; Zembala et al., 2010; Kumar et al., 2012; Gajewska et al., 2013) and SA (Hayat et al., 2010; Cui et al., 2012) for controlling heavy metal stress in different plants.

## Conclusions

Conclusively, Ni toxicity and deprivation of N, P, or K enhanced the production of ROS and caused lipid peroxidation thus, restricted the rice growth and mineral nutrient uptake. The negative effects of Ni toxicity were more with the interaction of N- or P- deprivation. However, seed priming of rice counteracted the Ni induced stress adversities in rice seedlings. The better growth and greater stress tolerance of primed rice seedlings was coordinately attributed to lower ROS production and accumulation, higher membrane stability, strong antioxidative defense system, and maintenance of mineral nutrient status.

## Data Availability Statement

The original contributions presented in the study are included in the article/supplementary material; further inquiries can be directed to the corresponding author.

## Author Contributions

FK: data curation, methodology, software, writing-original draft preparation. SH: conceptualization, methodology, supervision, writing—reviewing and editing. SK: software, writing—original draft preparation. MG: investigation, supervision, writing—reviewing and editing.

## Funding

The authors are thankful to Special Fund for Agro-scientific Research in the Public Interest of China (Project No. 201103005 & 201503122) for funding this study. 

## Conflict of Interest

The authors declare that the research was conducted in the absence of any commercial or financial relationships that could be construed as a potential conflict of interest.

## References

[B1] Ahmad I. Basra S. M. A. Hussain S. Hussain S. A. Rehman H. Rehman A. (2015). Priming with ascorbic acid, salicylic acid and hydrogen peroxide improves seedling growth of spring maize at suboptimal temperature. J. Environ. Agric. Sci. 3, 14–22

[B2] Athar R. Ahmad M. (2002). Heavy metal toxicity in legume microsymbiont system. J. Plant Nutr. 25, 369–386. 10.1081/PLN-100108842

[B3] Bailly C. Benamar A. Corbineau F. Dome D. (1996). Changes in malondialdehyde contents and in superoxide dismutase, catalase, glutathione reductase activities in sunflower seeds related to accelerated seed aging. Physiol. Plant 97, 104–110. 10.1111/j.1399-3054.1996.tb00485.x

[B4] Bailly C. El-Maarouf-Bouteau H. Corbineau F. (2008). From intracellular signaling networks to cell death: the dual role of reactive oxygen species in seed physiology. C. R. Biol. 331, 806–814. 10.1016/j.crvi.2008.07.022 18926495

[B5] Bal W. Kasprzak K. S. (2002). Induction of oxidative DNA damage by carcinogenic metals. Toxicol. Lett. 127, 55–62. 10.1016/S0378-4274(01)00483-0 12052641

[B6] Brune A. Deitz K. J. (1995). A comparative analysis of element composition of roots and leaves of barley seedlings grown in the presence of toxic cadmium, molybdenum, nickel and zinc concentrations. J. Plant Nutr. 18, 853–868. 10.1080/01904169509364943

[B7] Chen C. Huang D. Liu J. (2009). Functions and toxicity of nickel in plants: recent advances and future prospects. Clean. 37, 304–313. 10.1002/clen.200800199

[B8] Chen W. Guo C. Hussain S. Zhu B. Deng F. Xue Y. (2016). Role of xylo-oligosaccharides in protection against salinity-induced adversities in Chinese cabbage. Environ. Sci. Pollut. Res. 23, 1254–1264. 10.1007/s11356-015-5361-2 26358207

[B9] Cui W. Li L. Gao Z. Wu H. Xie Y. Shen W. (2012). Haem oxygenase-1 is involved in salicylic acid-induced alleviation of oxidative stress due to cadmium stress in Medicago sativa. J. Exp. Bot. 63, 695–709. 10.1093/jxb/ers201 22915740 PMC3444266

[B10] Duarte B. Delgado M. Ca ador I. (2008). The role of citric acid in cadmium and nickel uptake and translocation, in Halimione portulacoides. Chemosphere. 69, 836–840. 10.1016/j.chemosphere.2007.05.007 17585999

[B11] Foyer C. H. Noctor G. (2005). Oxidant and antioxidant signaling in plants: a re-evaluation of the concept of oxidative stress in a physiological context. Plant Cell. Environ. 28, 1056–1071. 10.1111/j.1365-3040.2005.01327.x

[B12] Gajewska E. Sklodowska M. (2005). Antioxidative responses and proline level in leaves and roots of pea plants subjected to nickel stress. Acta Physiol. Plant 27, 329–339. 10.1007/s11738-005-0009-3

[B13] Gajewska E. Sklodowska M. (2007). Effect of nickel on ROS content and antioxidative enzyme activities in wheat leaves. Bio. Metals. 20, 27–36. 10.1007/s10534-006-9011-5 16752220

[B14] Gajewska E. Sklodowska M. Slaba M. Mazur J. (2006). Effect of nickel on antioxidative enzyme activities, proline and chlorophyll contents in wheat shoots. Biol. Plant 50, 653–659. 10.1007/s10535-006-0102-5

[B15] Gajewska E. Wielanek M. Bergier K. Skłodowska M. (2009). Nickel- induced depression of nitrogen assimilation in wheat roots. Acta Physiol. Plant 31, 1291–1300. 10.1007/s11738-009-0370-8

[B16] Gajewska E. Drobik D. Wielanek M. Sekulska-Nalewajko J. Gocławski J. Mazur J. (2013). Alleviation of nickel toxicity in wheat (Triticum aestivum L.) seedlings by selenium supplementation. Biological Lett. 50 (2), 63–76.

[B17] Gajewska E. Drobik D. Wielanek M. Sekulska-Nalewajko J. Gocławski J. Mazur J. Skłodowska M. (2013). Alleviation of nickel toxicity in wheat (*Triticum aestivum* L.) seedlings by selenium supplementation. Biological Lett. 50 (2), 63–76.

[B18] Galan A. García-Bermejo L. Troyano A. Vilaboa N. E. Fernández C. de Blas E. Aller P. (2001). The role of intracellular oxidation in death induction (apoptosis and necrosis) in human promonocytic cells treated with stress inducers (cadmium, heat, X- rays). Eur. J. Cell Biol. 80, 312–320. 10.1078/0171-9335-00159 11370746

[B19] Gill S. S. Tuteja N. (2010). Reactive oxygen species and antioxidant machinery in abiotic stress tolerance in crop plants. Plant Physiol. Biochem. 48, 909–930. 10.1016/j.plaphy.2010.08.016 20870416

[B20] Gunes A. Inal A. Alpaslan M. Eraslan F. Bagci E. G. Cicek N. (2007). Salicylic acid induced changes on some physiological parameters symptomatic for oxidative stress and mineral nutrition in maize (Zea mays L.) grown under salinity. J. Plant Physiol. 164, 728–736. 10.1016/j.jplph.2005.12.009 16690163

[B21] Hao F. Wang X. Chen J. (2006). Involvement of plasma-membrane NADPH oxidase in nickel- induced oxidative stress in roots of wheat seedlings. Plant Sci. 170, 151–158. 10.1016/j.plantsci.2005.08.014

[B22] Hasanuzzaman M. Fujita M. (2011). Selenium pretreatment up regulates the antioxidant defense and methylglyoxal detoxification system and confers enhanced tolerance to drought stress in rapeseed seedlings. Biol. Trace Element Res. 143, 1758–1776. 10.1007/s12011-011-8998-9 21347652

[B23] Hasanuzzaman M. Anwar Hossain M. Fujita M. (2010). Selenium in higher plants: physiological role, antioxidant metabolism and abiotic stress tolerance. J. Plant Sci. 5, 354–375. 10.3923/jps.2010.354.375

[B24] Hayat Q. Hayat S. Irfan M. Ahmad A. (2010). Effect of exogenous salicylic acid under changing environment: A review. Environ. Exp. Bot. 68, 14–25. 10.1016/j.envexpbot.2009.08.005

[B25] Hussain S. Zheng M. Khan F. Khaliq A. Fahad S. Peng S. Huang J. Cui K. Nie L. (2015). Benefits of rice seed priming are offset permanently by prolonged storage and the storage conditions. Sci. Rep. 5 (1), 1–12. 10.1038/srep08101 PMC4309961 25631923

[B26] Hussain S. Khan F. Hussain H. A. Nie L. (2016a). Physiological and biochemical mechanisms of seed priming-induced chilling tolerance in rice cultivars. Front. Plant Sci. 7:116. 10.3389/fpls.2016.00116 26904078 PMC4746480

[B27] Hussain S. Khan F. Cao W. Wu L. Geng M. (2016b). Seed priming alters the production and detoxification of reactive oxygen intermediates in rice seedlings grown under sub-optimal temperature and nutrient supply. Front. Plant Sci. 7, 439. 10.3389/fpls.2016.00439 27092157 PMC4820636

[B28] Hussain S. Yin H. Peng S. Khan F. A. Khan F. Sameeullah M. (2016c). Comparative transcriptional profiling of primed and non-primed rice seedlings under submergence stress. Front. Plant Sci. 7, 1125. 10.3389/fpls.2016.01125 27516766 PMC4964843

[B29] Hussain S. Khaliq A. Tanveer M. Matloob A. Hussain H. A. (2018). Aspirin priming circumvents the salinity-induced effects on wheat emergence and seedling growth by regulating starch metabolism and antioxidant enzyme activities. Acta Physiol. Plant 40, 68. 10.1007/s11738-018-2644-5

[B30] Hussain S. Khaliq A. Noor M. A. Tanveer M. Hussain H. A. Hussain S. (2020a). “Metal Toxicity and Nitrogen Metabolism in Plants: An Overview,” in Carbon and Nitrogen Cycling in Soil (Singapore: Springer), 221–248.

[B31] Hussain H. A. Men S. Hussain S. Zhang Q. Ashraf U. Anjum S. A. (2020b). Maize tolerance against drought and chilling stresses varied with root morphology and antioxidative defense system. Plants 9, 720. 10.3390/plants9060720 PMC7356637 32517168

[B32] Jisha K. C. Vijayakumari K. Puthur J. T. (2013). Seed priming for abiotic stress tolerance: An overview. Acta Physiol. Planta. 35, 1381–1396. 10.1007/s11738-012-1186-5

[B33] Khaliq A. Aslam F. Matloob A. Hussain S. Geng M. Wahid A. (2015). Seed priming with selenium: consequences for emergence, seedling growth, and biochemical attributes of rice. Biol. Trace Elem. Res. 166, 236–244. 10.1007/s12011-015-0260-4 25690516

[B34] Khan F. Hussain S. Tanveer M. Khan S. Hussain H. A. Iqbal B. (2018). Coordinated effects of lead toxicity and nutrient deprivation on growth, oxidative status, and elemental composition of primed and non-primed rice seedlings. Environ. Sci. Pollut. Res. 25, 21185–21194. 10.1007/s11356-018-2262-1 29774513

[B35] Kukkola E. Rautio P. Huttunen S. (2000). Stress indications in copper- and nickel-exposed Scots pine seedlings. Environ. Exp. Bot. 43, 197–210. 10.1016/S0098-8472(99)00057-X 10725519

[B36] Kumar P. Tewari R. K. Sharma P. N. (2007). Excess nickel-induced changes in antioxidative processes in maize leaves. J. Plant Nutr. Soil Sci. 170, 796–802. 10.1002/jpln.200625126

[B37] Kumar M. Bijo A. J. Baghel R. S. Reddy C. R. K. Jha B. (2012). Selenium and spermine alleviate cadmium induced toxicity in the red seaweed *Gracilaria dura* by regulating antioxidants and DN A methylation. Plant Physiol. Biochem. 51, 129–138. 10.1016/j.plaphy.2011.10.016 22153249

[B38] Miller G. A. D. Suzuki N. Ciftci-Yilmaz S. Mittler R. O. N. (2010). Reactive oxygen species homeostasis and signalling during drought and salinity stresses. Plant Cell Environ. 33, 453–467. 10.1111/j.1365-3040.2009.02041.x 19712065

[B39] Ouzounidou G. Moustakas M. Symeonidis L. Karataglis S. (2006). Response of wheat seedlings to Ni stress: Effects of supplemental calcium. Arch. Environ. Contam. Toxicol. 50, 346–352. 10.1007/s00244-005-5076-3 16362494

[B40] Pandey N. Sharma C. P. (2002). Effect of heavy metals Co2+, Ni2+ and Cd2+on growth and metabolism of cabbage. Plant Sci. 163, 753–758. 10.1016/S0168-9452(02)00210-8

[B41] Papadopoulos A. Prochaska C. Papadopoulos F. Gantidis N. Metaxa E (2007). Determination and evaluation of cadmium, copper, nickel, and zinc in agricultural soils of western Macedonia, Greece. Environ. Manage. 40, 719–726. 10.1007/s00267-007-0073-0 17879129

[B42] Patterson B. D. MacRae E. A. Ferguson I. B. (1984). Estimation of hydrogen peroxide in plant extracts using titanium (IV). Anal. Biochem. 139, 487–492. 10.1016/0003-2697(84)90039-3 6476384

[B43] Pillay S. V. Rao V. S. Rao K. V. N. (1996). Effect of nickel toxicity in *Hyptis suareeolens* (L.) Poit and Helianthus annuus L. Indian J. Plant Physiol. 1, 153–156.

[B44] Rao K. V. M. Sresty T. V. (2000). Antioxidative parameters in the seedlings of pigeonpea (*Cajanus cajan* (L.) Millspaugh) in response to Zn and Ni stresses. Plant Sci. 157, 113–128. 10.1016/S0168-9452(00)00273-9 10940475

[B45] Rizwan M. Mostofa M. G. Ahmad M. Z. Zhou Y. Adeel M. Mehmood S. (2019). Hydrogen sulfide enhances rice tolerance to nickel through the prevention of chloroplast damage and the improvement of nitrogen metabolism under excessive nickel. Plant Physiol. Biochem. 138, 100–111. 10.1016/j.plaphy.2019.02.023 30856414

[B46] Salt D. E. Prince R. C. Pickering I. J. Raskin I. (2000). In Phytoremediation of Contaminated Soil and Water. Eds. Terry N. Banuelos G. (Boca Raton, FL: Lewis Publishers), 189–200.

[B47] Shah H. Jalwat T. Arif M. Miraj G. (2012). Seed priming improves early seedling growth and nutrient uptake in mungbean. J. Plant Nutr. 35, 805–816. 10.1080/01904167.2012.663436

[B48] Shah Z. Haq I. U. Rehman A. Khan A. Afzal M. (2013). Soil amendments and seed priming influence nutrients uptake, soil properties, yield and yield components of wheat (*Triticum aestivum* L.) in alkali soils. Soil Sci. Plant Nutr. 59 (2), 262–270. 10.1080/00380768.2012.762634

[B49] Shin R. Berg R. H. Schachtman D. P. (2005). Reactive oxygen species and root hairs in Arabidopsis root response to nitrogen, phosphorus and potassium deficiency. Plant Cell Physiol. 46, 1350–1357. 10.1093/pcp/pci145 15946982

[B50] Sun H. W. Ha S. J. Liang X. Kang W. J. (2010). Protective role of selenium on garlic growth under cadmium stress. Commun. Soil Sci. Plant Anal. 41, 1195–1204. 10.1080/00103621003721395

[B51] Walter J. Jentsch A. Beierkuhnlein C. Kreyling J. (2012). Ecological stress memory and cross stress tolerance in plants in the face of climate extremes. Environ. Exp. Bot. 94, 3–8. 10.1016/j.envexpbot.2012.02.009

[B52] Yoshida S. Forno D. A. Cook J. H. Gomez K. A. (1976). Laboratory Manual for Physiological Studies of Rice. 3rd ed. (Los Banos, Philippines: The International Rice Research Institute), 61–66.

[B53] Zembala M. Filek M. Walas S. Mrowiec H. Kornas A. Miszalski Z. (2010). Effect of selenium on macro- and microelement distribution and physiological parameters of rape and wheat seedlings exposed to cadmium stress. Plant Soil. 329 (1-2), 457–468. 10.1007/s11104-009-0171-2

[B54] Zhao J. Shi G. Yuan Q (2008). Polyamines content and physiological and biochemical responses to ladder concentration of nickel stress in Hydrocharis dubia (Bl.) Backer leaves. BioMetals. 21, 665–674. 18587652 10.1007/s10534-008-9151-x

[B55] Zheng M. Tao Y. Hussain S. Jiang Q. Peng S. Huang J. (2016). Seed priming in dry direct- seeded rice: consequences for emergence, seedling growth and associated metabolic events under drought stress. Plant Growth Regul. 78, 167–178. 10.1007/s10725-015-0083-5

